# Analysis of bleeding after ultrasound-guided core needle biopsy of infected cervical lymph nodes

**DOI:** 10.3389/fsurg.2025.1589238

**Published:** 2025-08-08

**Authors:** Wenzhi Zhang, Dan Li, Dongming Su

**Affiliations:** Department of Ultrasonography, Hangzhou Red Cross Hospital (Integrated Chinese and Western Hospital of Zhejiang Province), Hangzhou, Zhejiang, China

**Keywords:** ultrasound, cervical, lymph nodes, infection, needle biopsy, bleeding

## Abstract

**Purpose:**

This study aimed to determine the incidence of bleeding after core needle biopsy (CNB) of infected cervical lymph nodes and analyze the factors associated with bleeding after CNB, and preventive measures of bleeding.

**Methods:**

We retrospectively analyzed the records of 643 patients with infectious cervical lymphatic ganglionic diseases who underwent CNB at our hospital from December 2015 to February 2022. The number of patients with bleeding after CNB, extent of bleeding, and type of disease were recorded and statistically analyzed.

**Results:**

A total of 643 patients with cervical lymph node infection were included in this study. The postoperative and intraoperative bleeding rate of CNB was 23.48% (150/643). Internal lymph node bleeding was most commonly reported (94.0%, 141/150). Lymph nodes containing pus had a higher risk of bleeding than solid lymph nodes (*χ*²: 12.00, P: 0.001). Lymph node tuberculosis had a significantly higher risk of bleeding than lymph node infection with common bacteria (*χ*²: 4.10, P: 0.04).

**Conclusion:**

Following CNB, patients with cervical lymph node infection primarily reported internal lymph node bleeding. Lymph nodes with an internal pus cavity surrounded by blood-rich granulation tissue showed a higher risk of bleeding than solid lymph nodes, with heterogeneous enhancement on preoperative contrast-enhanced ultrasound.

## 1 Introduction

Reactive hyperplasia, infection, lymphoma, and metastasis of malignant tumors are common causes of cervical lymphadenopathy. Reactive disease (52.2%) and granulomatous disease (15.5%) were the most common pathologic diagnoses in a study of cervical lymph node biopsies in the eastern region of Saudi Arabia (1). Determination of the nature of enlarged lymph nodes guides the development of treatment plans. For example, the clinical diagnosis of lymph node tuberculosis is difficult (2). Once diagnosed, antituberculosis drugs must be administered for more than 1 year (3). Moreover, its recurrence rate is high (4), especially after the emergence of drug-resistant strains, making treatment more difficult. Some infected lymph nodes can recover with only short-term treatment with sensitive antimicrobials. Some cases of Kikuchi–Fujimoto disease can even be treated without antimicrobial treatment. Therefore, safely obtaining effective tissue or abscess specimens for pathological examination, biological culture, and drug sensitivity assays in a minimally invasive manner is necessary. Currently, the application of contrast-enhanced ultrasound (CEUS)-assisted core needle biopsy (CNB) has gradually increased (5, 6). Several studies (7–10) have confirmed that the success rates of needle biopsy and postoperative pathological diagnosis are extremely high (83.8%–100%). A previous study reported the risk of bleeding after lymph node CNB, and the bleeding rate of malignant lymph nodes was approximately 0.1% (11). There is a lack of research on the risk of bleeding after ultrasound-guided CNB (US-CNB) in infected cervical lymph nodes, especially the lack of large sample size data to quantify the difference in bleeding risk in different infected types of lymph nodes. Therefore, this study aimed to determine the bleeding rate after US-CNB of infected cervical lymph nodes and provide a reference for preoperative evaluation and related studies on US-CNB of cervical lymph nodes.

## 2 Materials and methods

### 2.1 Patient selection

**Figure 1:**
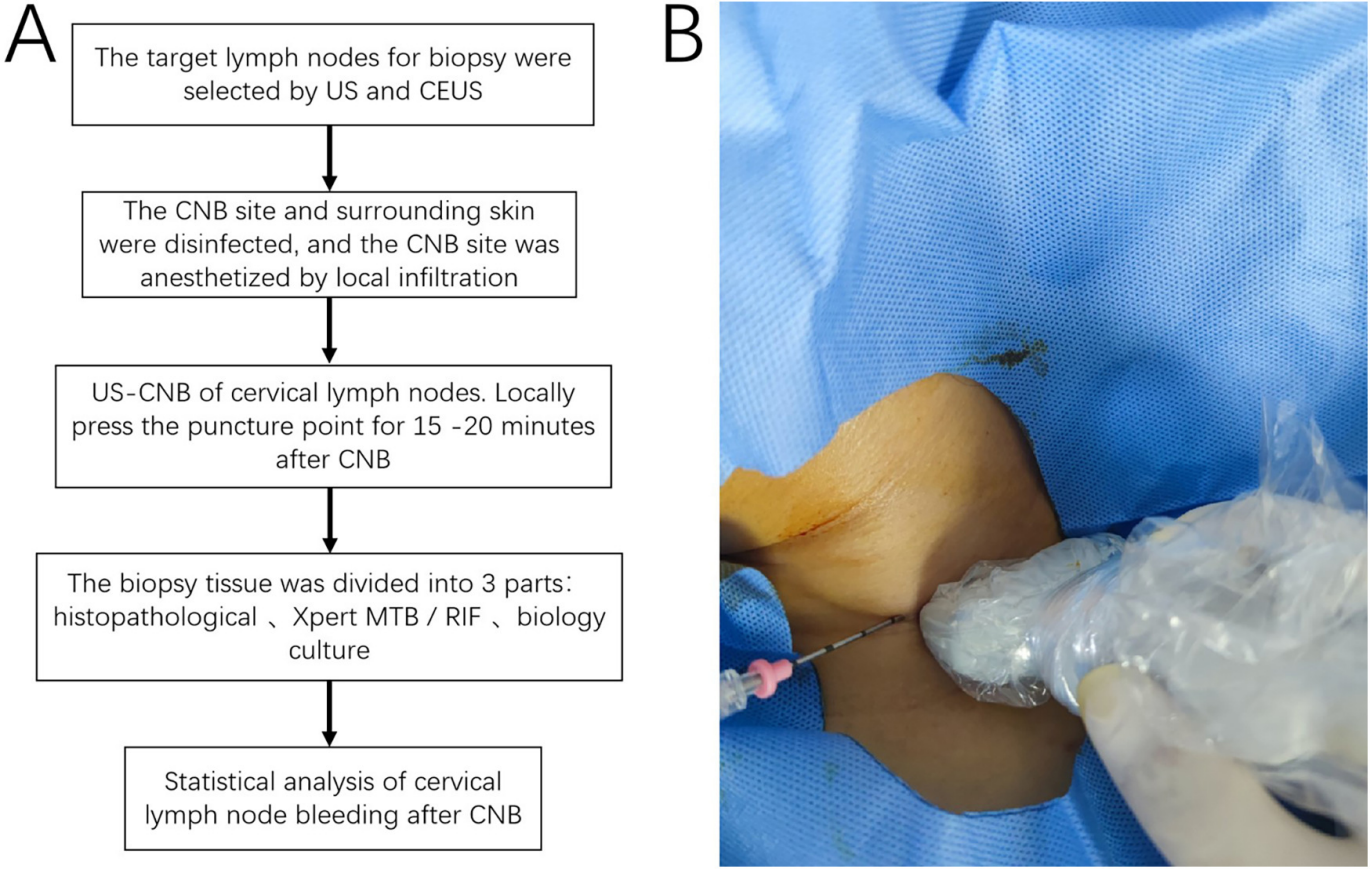
**(A)** Flowchart of the study, the procedure comprised target node selection, disinfection, biopsy, compression hemostasis, sample processing, and statistical analysis; **(B)** US-CNB procedure. CNB, core needle biopsy; US-CNB, ultrasound-guided core needle biopsy; MTB, *Mycobacterium tuberculosis* bacilli; RIF, rifampicin.

This study was reviewed and approved by the Ethics Committee of the Hangzhou Red Cross Hospital. Written informed consent was obtained from the patients, and the study was performed in accordance with the Declaration of Helsinki. We retrospectively analyzed 643 patients admitted to our hospital from December 2015 to February 2022 who underwent US examination and CEUS and US-CNB of infected cervical lymph nodes (Figure 1). All patients were suspected to have infectious lymph nodes through laboratory examination and imaging examination by clinicians, combined with clinical symptoms, CNB pathological examination, bacterial culture, Xpert Mycobacterium Tuberculosis/Rifampicin (Xpert MTB/RIF) assay of diseased lymph nodes, etc., to find pathogenic bacteria, and drug sensitivity test was carried out to guide clinical treatment. All patients with infectious lymph nodes included in the study were confirmed by surgery or CNB pathology, bacterial culture, and Xpert MTB/RIF assay. Bleeding during and after US-CNB was determined via US exploration as an anechoic or hyperechoic zone around and inside the lymph nodes as follows: (1) Minor bleeding: crescent-shaped bleeding around the lymph nodes with no significant increase in lymph node volume compared with that before CNB. (2) Moderate bleeding: (a) bleeding in the lymph nodes after CNB results in increased lymph node volume; (b) extensive perilymph node bleeding after CNB results in lymph node displacement and deformation (3) Massive bleeding: lymph nodes surrounded by hematoma or lymph nodes squeezed and displaced, necessitating surgical treatment and on-site rescue (e.g., sudden appearance of a lump at the piercing site, large ecchymosis on the skin of the piercing area, sudden drop in blood pressure). The abovementioned extent of bleeding is based on the study conducted by Zhang et al. (12). Three attending physicians with >5 years of lymph node CNB experience assessed the extent of bleeding in all patients to correct for study bias.

The inclusion criteria were as follows: (1) cervical lymph nodes with a diameter of >1.0 cm and maximum short diameter of >0.5 cm; (2) age >18 years; (3) normal blood clotting function; (4) normal heart and lung function; and (5) clinically suspected infectious lesions based on laboratory findings.

The exclusion criteria were as follows: (1) patients with a history of food allergy; (2) those who took anticoagulant drugs within the past week; (3) those with a history of mental illness; (4) those with skin ulcers and severe psoriasis at the puncture site; (5) those with lymph nodes adjacent to major blood vessels in which bleeding and other complications during and after biopsy are unavoidable; and (6) those with clinically suspected malignant lymph nodes based on examination results.

### 2.2 US and CEUS examination

A Philips iU22® ultrasound machine (Philips, Amsterdam, The Netherlands) was used with L12-5 and an L9-3 probes. During the examination, the patient was placed in a comfortable position, and their head was fixed with a cervical pillow. The size, distribution, internal echo, and activity of the cervical lymph nodes as well as the integrity of the capsule were assessed via routine US. The relationship between the lymph nodes and large cervical vessels was monitored to avoid massive postoperative bleeding. According to the comparison between the lymph nodes and the surrounding tissues, the echo in the lymph nodes is higher than that in the surrounding tissues, and the lymph nodes show hyperecho. If there is no echo reflex in the lymph nodes, it shows anechoic.

SonoVue was used after dilution with 5 ml of normal saline, and the mixture was shaken well. Next, 2.4 ml of the mixture was injected through a superficial vein of the elbow. Finally, 5 ml of normal saline was injected into the flushing tube. A low mechanical index of 0.06 was used for the entire imaging process. Uniform filling of contrast agent in lymph nodes is called homogeneous enhancement, while non-uniform filling of contrast agent in lymph nodes is called Heterogeneous enhancement. The time button and the dynamic storage button were pressed simultaneously with contrast agent injection to observe its perfusion into the selected lymph node. Using CEUS, the enhancement mode and range of the lymph nodes were determined.

### 2.3 US-CNB

The target lymph node—the largest lymph node or a lymph node with abnormalities—was selected based on the results of US and CEUS examinations. To improve safety, CNB was performed using a semiautomatic biopsy gun (18G, TSK, Japan). All cervical lymph nodes were biopsied using US-CNB. Three specimens were collected from different directions. One specimen was fixed with 10% formaldehyde and sent to the laboratory for pathological examination. The other two specimens were placed in sterile containers and sent to the laboratory for biological culture and Xpert MTB/RIF assay, respectively. Intra- and postoperative echogenic changes in and around the lymph nodes, hyperechogenic zones, and anechogenic zones were observed, and their ranges were measured. Postoperative compression was applied to stop the bleeding for 15–20 min. The observation times were as follows: during the procedure, after the procedure, 1 h after the procedure, and 24 h after the procedure. US primarily revealed the presence or absence of bleeding in and around the lymph nodes. Lymph node bleeding was defined as the presence of anechoic or hyperechoic zones in or around the lymph nodes. To ensure the reliability of the results, all CNBs are performed by two sonographers with more than three years of puncture and diagnosis experience. Two independent sonographers, blinded to each other's assessments, evaluated bleeding outcomes. When the two sonographers had different opinions, a third sonographer with more than five years of experience gave the final result after further data analysis.

### 2.4 Statistical analysis

The data obtained were analyzed using Statistical Package for the Social Sciences, version 19.0 (IBM Corp., Armonk, NY, USA). categorical data for the number of patients with bleeding after CNB and the type of disease were analyzed using the chi-square test and Fisher's exact test. *P*-values of <0.05 were considered to indicate statistical significance.

## 3 Results

### 3.1 Patient clinical characteristics and US and CEUS features

Among the 643 enrolled patients, included 238 males and 405 females, with an average age of 32.34 ± 5.18 (range, 18–66) years. The duration of the disease ranged from 3 to 123 (mean: 41.34 ± 10.3) days. The clinical symptoms of patients and CEUS patterns of the target lymph nodes are shown in Table 1.

**Table 1:** Clinical symptoms, lymph node distribution, lymph node enhancement pattern.

Clinical features	No. of patients (*n*)	Percentage (%)
Lymph node distribution		
Bilateral	170	26.4
Unilateral	473	73.6
No. of lesions		
Single	32	5.0
Multiple	611	95.0
Pain and tenderness		
Yes	125	19.4
No	518	80.6
Mobility of lymph node		
Well	78	12.1
Poor	565	87.9
History of tuberculosis		
Yes	334	51.9
No	309	48.1
CEUS mode		
Homogeneous enhancement	52	8.1
Heterogeneous enhancement	591	91.9

Among the 643 patients, 73 had common bacterial lymphadenitis, 13 had fungal lymphadenitis, 496 had nodal tuberculosis, 56 had nontuberculous mycobacterial lymphadenitis, and 5 had viral lymphadenitis. Moreover, 591 patients had heterogeneous enhancement and 52 had homogeneous enhancement before US-CNB. Enhancement from the lymphatic hilum to the periphery was observed in 402 cases, diffuse enhancement was observed in 210 cases, and centripetal enhancement was noted in 31 cases.

### 3.2 Relationship between bleeding and disease after CNB

The following results demonstrate the bleeding incidence patterns and their relationship with lymph node characteristics identified through our methodological approach. The types of lymph node diseases and bleeding after US-CNB are presented in Table 2.

**Table 2:** Types of lymph node diseases and incidence of bleeding after US-CNB.

Types of lymph node diseases	No. of patients (*n*)	Bleeding cases (*n*)	Bleeding rate (%)
Common bacterial infection (73)		10	13.69
Streptococcal infection	53	10	18.87
Staphylococcal infection	7	1	14.28
Others	13	0	0
Fungal infection (13)		0	0
Tuberculous infection (496)		121	24.39
Non-tuberculous mycobacterial infection (56)		8	14.28
Virus infection (5)		0	0
Total	643	150	23.48

Note: The bleeding rate after US-CNB of common lymph node infection and lymph node tuberculosis was compared, *χ*² was 4.10, *P* was 0.04. The bleeding rate after US-CNB of Non-tuberculous mycobacterial infection and lymph node tuberculosis was compared, *χ*² was 2.872, *P* was 0.09, no statistical significance.

**Figure 2:**
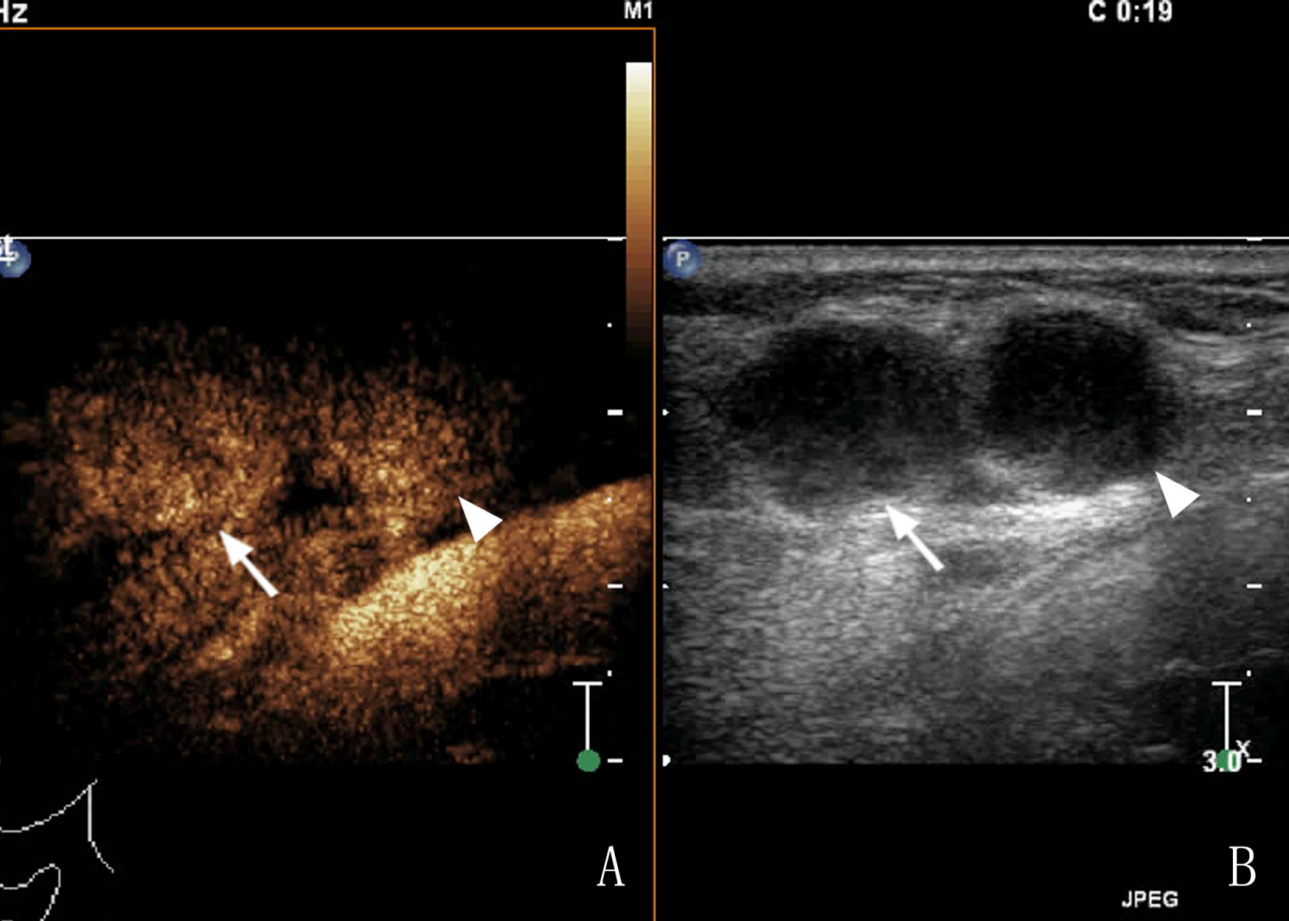
A 65-year-old female patient had cervical LN TB, **(A)** Two lymph nodes (arrows) showed homogeneous enhancement in CEUS; **(B)** routine US; the arrow and arrow head shows a tuberculous lymph node.

**Figure 3:**
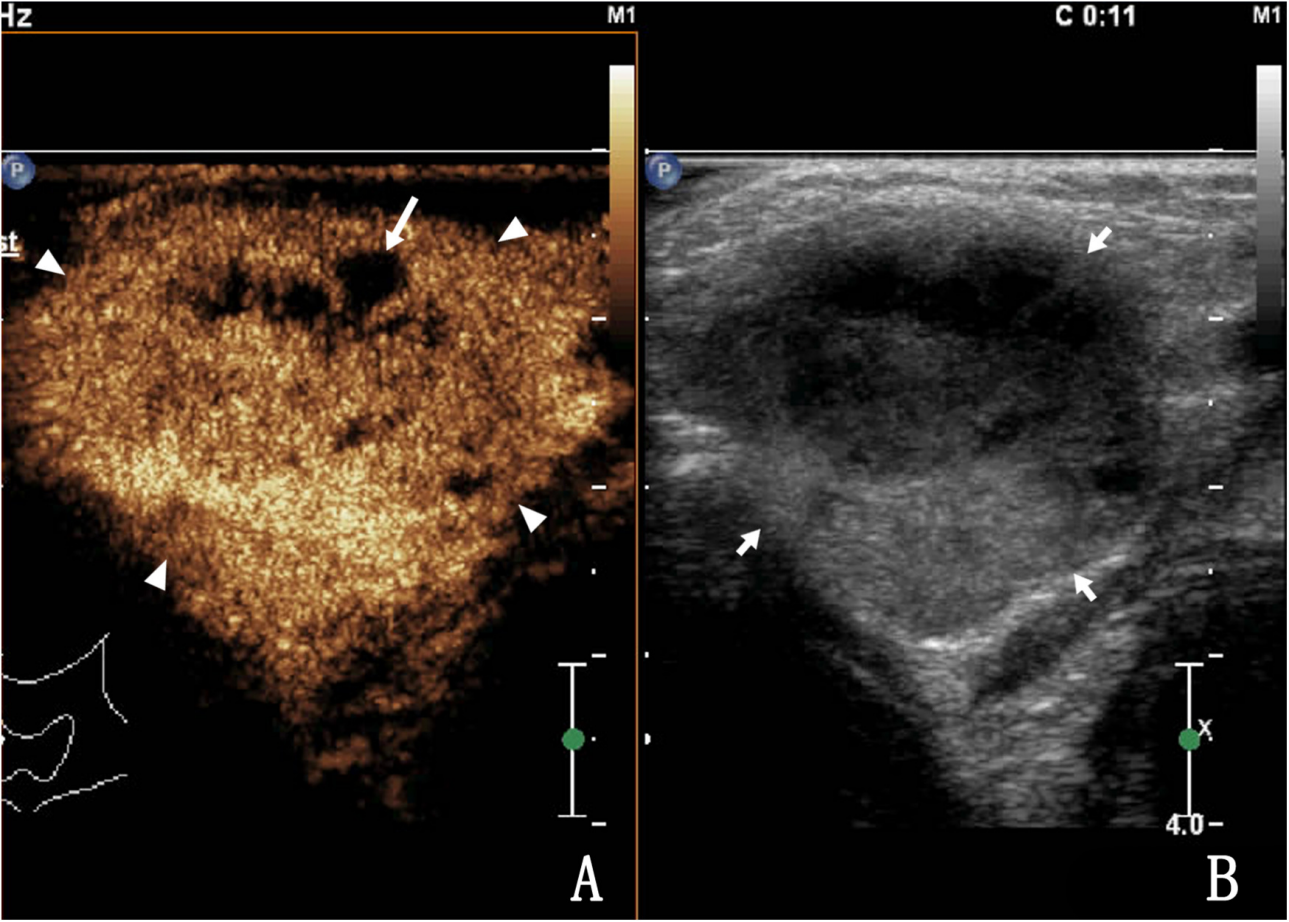
A 34-year-old female with lymph node tuberculosis of the right cervical lymph node. **(A)** CEUS revealed heterogeneous enhancement, with unenhanced areas, which were liquefaction of necrotic areas (arrows). The surrounding tissues showed circular enhancement (triangular arrows) resulting from congestion and edema. The enhanced area was larger than that observed on routine US. **(B)** Routine US; the arrow shows a tuberculous lymph node.

Of the 150 patients with bleeding in and around the lymph nodes after US-CNB, lymph node mobility on palpation showed no statistically significant association with bleeding after CNB. Lymph nodes containing pus (heterogeneous enhancement) had a higher risk of bleeding than solid lymph nodes (homogeneous enhancement) (*χ*²: 12.00, P: 0.001; Table 3). Of the 150 patients, 56 had high enhancement, 94 had low enhancement, 2 had homogeneous enhancement (Figure 2), and 148 had heterogeneous enhancement (Figure 3).

**Table 3:** Relationship between palpation lesion lymph node activity, pus—containing lymph nodes (heterogeneous enhancement) and bleeding after US-CNB (*n*).

Lymph node features	Bleeding (150)	No bleeding (493)	*x* ^2^	*P*
Movable (78)	18	60	0.03	0.95
Immobile (565)	132	433		
Purulent cavity (591)	148	443	12.00	0.001
Solid lymph node (52)	2	50		

Note: Purulent cavity: contrast-enhanced ultrasound in the lymph nodes showed patchy but no enhancement, and aspiration revealed pus; solid lymph node: lymph nodes showing homogeneous enhancement on contrast-enhanced ultrasound.

**Figure 4:**
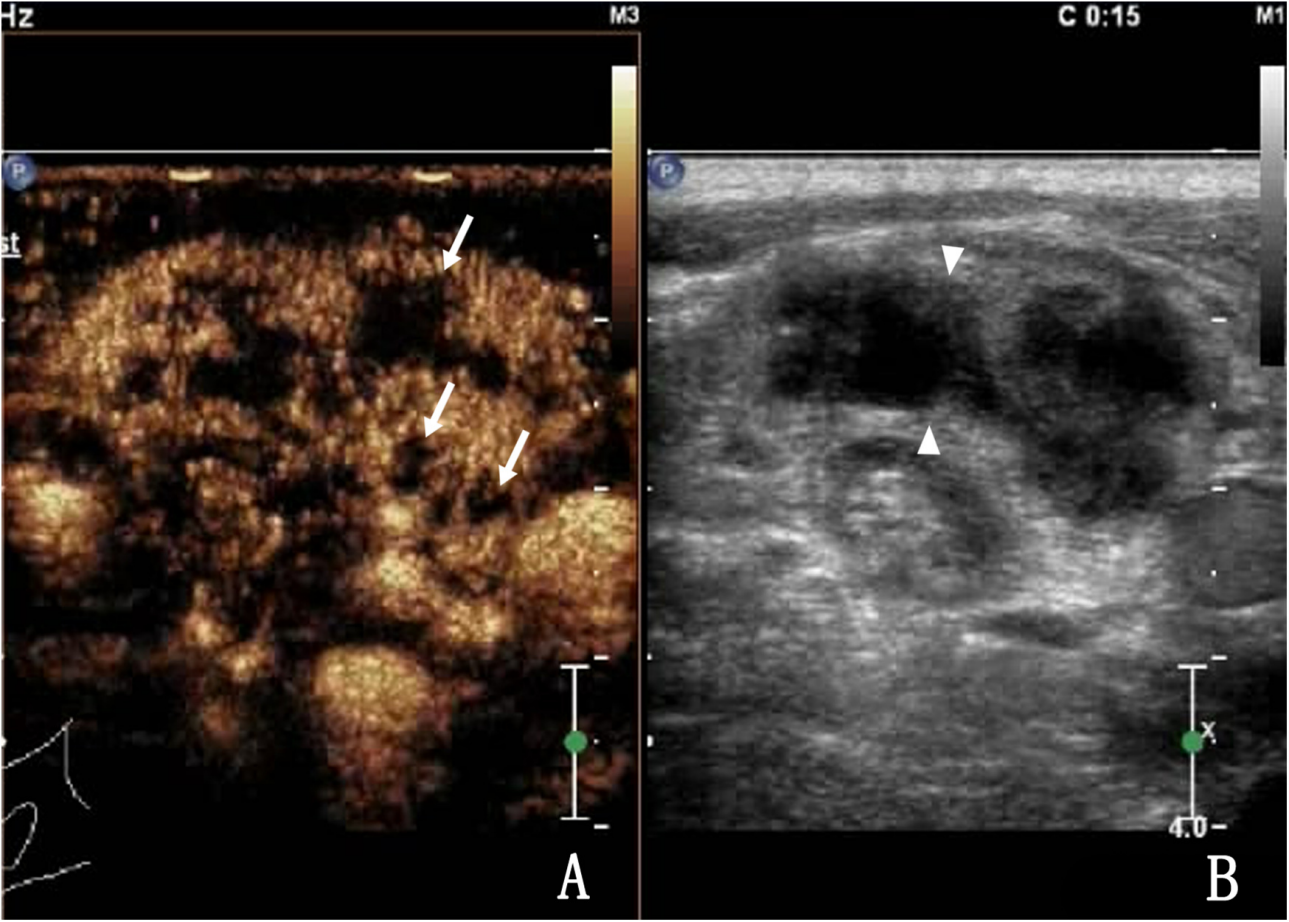
A 27-year-old male with lymph node tuberculosis of the right cervical lymph node. **(A)** CEUS showed heterogeneous enhancement, with multiple unenhanced areas exhibiting irregular shapes, which are liquefaction necrotic areas, also known as abscess cavities (arrow). **(B)** Routine US; lymph nodes fused to each other (triangular arrow).

**Figure 5:**
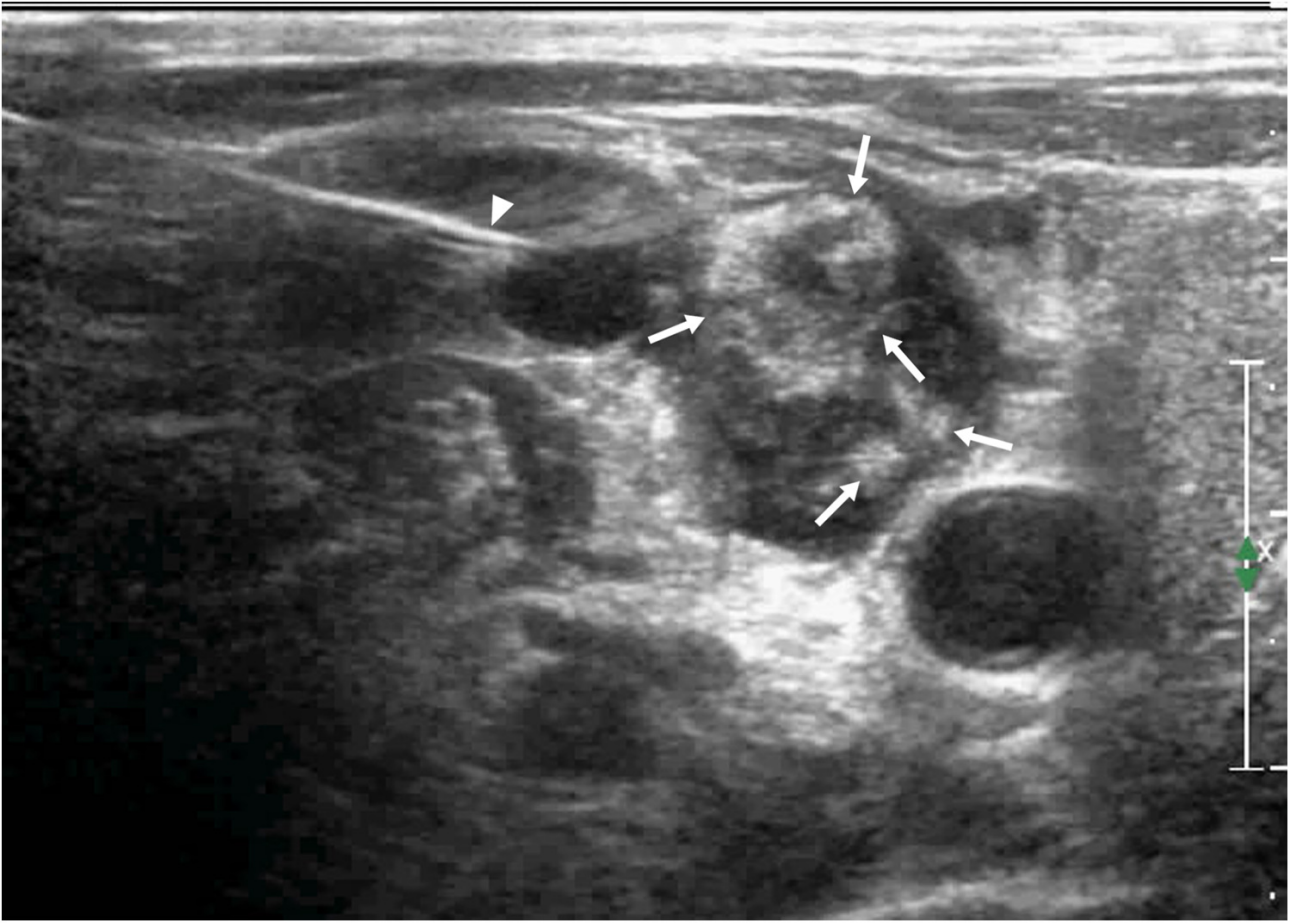
The same patient as shown in Figure 4. During the second needle entry of US-CNB, the triangle arrow shows the core needle, bleeding was observed in the lymph node, and the abscess cavity was filled with fresh blood (arrow).

**Figure 6:**
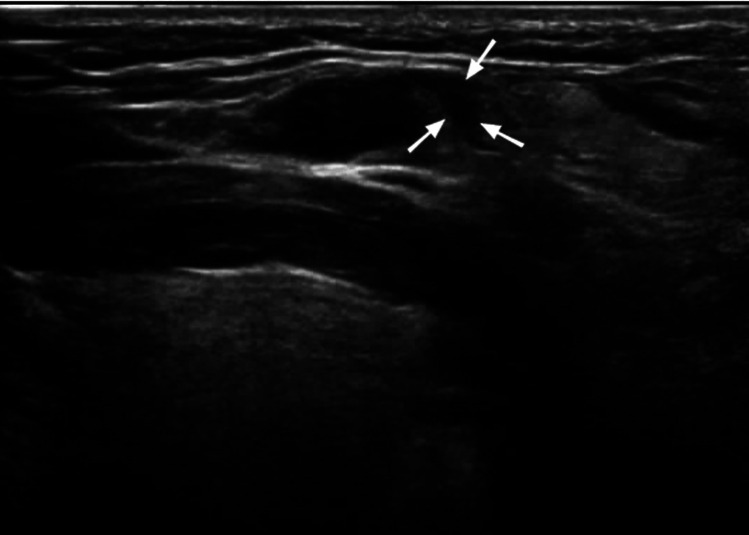
A 32-year-old female patient with cervical LN TB had crescent-shaped bleeding around the lymph nodes after two puncture biopsies (arrows).

There was minor bleeding after biopsy in 150 patients, and no patients had large hematomas or bleeding that required surgery. Among them, 141 patients had internal lymph node bleeding (Figures 4, 5), 7 had bleeding in and around the lymph nodes, and 2 had bleeding around the lymph nodes (Figure 6). The main parameter for minor bleeding in the lymph nodes was whether there was a high echo in the lymph nodes, whether the volume of the lymph nodes decreased after pus pumping, or whether the volume of the lymph nodes increased during and after CNB. The main parameter for slight bleeding around the lymph nodes was whether there was crescent anechosis. The observation time for the abovementioned lymph node bleeding was immediately, 1 h, and 24 h after the procedure.

## 4 Discussion

With the recent development of US imaging technology, its specificity and sensitivity for the internal structures of lymph nodes have significantly improved (8, 13), and the detection rate of lymph node enlargement has increased.

Currently, US signs of cervical lymph node structural abnormalities include increased lymph node size and cortical thickness, morphological changes, changes in the internal echo (including necrosis and calcification), destruction and disappearance of the lymphatic hilum, and capsule rupture (14, 15). CEUS of infected lymph nodes often reveals a heterogeneous enhancement (16). When an abscess is formed, the lymph nodes show unenhanced areas; however, these enhanced areas may vary depending on the course of the disease and the source of infection.

The gold standard for the diagnosis of infectious lymph node diseases is pathological examination and biological culture (17); therefore, imaging cannot replace pathological examination. Currently, US-CNB facilitates rapid acquisition of pathological and biological culture specimens and is widely used in clinical practice because it is a rapid and minimally invasive procedure.

Kim et al. (18) retrospectively evaluated the sensitivity, specificity, and accuracy of US-CNB for diseased cervical lymph nodes, which were 97%, 99%, and 97.9%, respectively. Regarding recent studies on CNB of neck masses, there have been few reports of serious complications, and post-procedure bleeding is the most common complication, with an incidence of 0.8%–3.0% (19, 20). In the present study, the rate of intra-procedure and post-procedure bleeding in US-CNB of infected cervical lymph nodes was 23.48%. This may be related to the type of disease we choose, our hospital is a regional center for tuberculosis diagnosis and treatment. We found that patients with infectious lymphadenitis were prone to internal lymph node bleeding during CNB, which was related to heterogeneous enhancement on CEUS. In this study, 148 of the 150 patients with hemorrhage showed inhomogeneous enhancement. The infected lymph nodes exhibited heterogeneous enhancement, and areas of liquefaction and necrosis were observed, which are commonly known as abscess cavities. During CNB, the blood in the needle passage penetrates into the abscess cavities, leading to bleeding in the lymph nodes. We believe that this is related to the granuloma with abundant blood supply around the pus cavity (16). After CNB of the granuloma, the blood in the needle passage enters the pus cavity in the low-pressure area. In some patients with infectious lymph node diseases, such as those with suspected cervical lymph node tuberculosis, the abscess cavity puncture fluid is used for testing (21). After aspirating the puncture fluid, the abscess cavity pressure decreases, creating a low-pressure area. Performing CNB in this cavity causes bleeding that temporarily increases the pressure. This bleeding typically stops once pressure equilibrates. Among our 150 bleeding cases, 141 (94%) showed internal lymph node bleeding.

With disease progression, infection of the connective tissues surrounding the lymph nodes often results in adhesion. Thus, CEUS revealed annular and heterogeneous enhancement around the target lymph nodes (16, 22, 23). Adhesions lead to poor lymph node mobility during clinical palpation, which could be easily misdiagnosed as malignant lymph nodes. CNB is often used for differential diagnosis. In the present study, the lymph nodes adhered to the surrounding area during and after CNB with poor lymph node mobility. Bleeding around the target lymph node is unlikely; however, there is a high risk of internal bleeding. We hypothesized that in infectious lymph nodes, surrounding tissue capillary expansion, organization hyperemia, and increased lymph node volume enhance the pressure of the surrounding tissue, which increases the risk of bleeding after a biopsy of the surrounding tissue. During postoperative oppression hemostasis, the lateral pressure of the lymph nodes is higher than that of the abscess cavity, directing the flow of bleeding toward the abscess cavity; however, further studies are needed to confirm this view. Furthermore, patients with infectious lymph nodes and prominent clinical symptoms, such as redness, swelling, heat, and pain, may experience pain during oppression hemostasis, particularly if the operator fails to properly apply compression, which may lead to bleeding.

In this study, CEUS revealed that lymph nodes with homogeneous enhancement had a lower bleeding rate than those with heterogeneous enhancement, which may be related to lymphocyte proliferation, vasodilatation of infectious lymph nodes (16), increased volume of the internal structure, and increased internal pressure under the limitation of the lymph node capsule, which caused the lymph nodes to appear round or oval (23). Following US-CNB, the high-pressure area around the needle tract quickly provides a natural pressure to the needle tract, and this compression hemostasis after US-CNB greatly reduces the probability of bleeding in the lymph nodes.

Homogeneously enhanced lymph nodes do not form abscesses. This often indicates that during the initial stage of infection, edema and telangiectasia of the surrounding tissues are not obvious, and the probability of bleeding around the lymph nodes is low. In the present study, of the 52 patients with homogeneously enhanced infectious lymph nodes, two experienced bleeding after CNB and both showed minor bleeding around the lymph nodes.

The observed bleeding rates were 18.87% in lymph node tuberculosis vs. 13.69% in lymph node infection with common bacteria. This discrepancy may be attributed to earlier diagnosis and intervention in bacterial infections due to acute symptoms (pain, fever), which potentially limits the progression of intranodal neoangiogenesis and hypervascularity. In contrast, granulomatous infections (tuberculosis) often present insidiously, allowing prolonged vascular proliferation that may increase bleeding susceptibility during biopsy (12). The bleeding rate of CNB in lymph node tuberculosis was higher, but there was no statistical significance, which may be related to the insufficient number of nontuberculosis mycobacterium cases.

It is necessary to perform CEUS of target lymph nodes before CNB. CEUS indicated the presence of infected lymph nodes, with the granuloma enveloping abscesses. The operator should conduct timely and accurate pressure hemostasis in the interval between the two CNBs. If bleeding is substantial, surgical intervention may be required (14).

This study has several limitations. Firstly, its retrospective single-center design may lead to case selection bias and limit the external validity of the research results. Secondly, the analyzed infectious lymph nodes mainly originated from tuberculosis and did not fully represent other types of infections (such as bacterial and fungal). Therefore, the reported bleeding rate may not accurately reflect the overall risk of all infectious lymph nodes after US-CNB. Thirdly, performing puncture operations with only a single type of biopsy needle cannot assess the potential impact of different biopsy needle models on the risk of bleeding. Future research urgently needs: (1) to incorporate more diverse types of infectious lymph nodes and larger sample sizes, or preferably conduct prospective multicenter studies. Multicenter design can incorporate a broader patient population, significantly enhance the external validity of research conclusions, and more accurately assess the bleeding risk of different types of infectious lymph nodes. (2) Compare the application of different types of biopsy needles in US-CNB of infectious lymph nodes to accurately quantify the bleeding risk of different needles and provide evidence-based basis for the best selection of puncture tools in clinical practice.

## 5 Conclusion

The incidence of bleeding in the diagnosis of infectious lymph nodes by US-CNB was 23.48% (mainly mild intraducosal bleeding). Heterogeneous CEUS enhancement and abscess cavities increased bleeding risk. Limitations include single-center design and TB case predominance; future multicenter studies should validate findings and compare biopsy needle types.

## Data Availability

The original contributions presented in the study are included in the article/Supplementary Material, further inquiries can be directed to the corresponding author.
